# RNA-Seq Analysis Reveals a Negative Role of KLF16 in Adipogenesis

**DOI:** 10.1371/journal.pone.0162238

**Published:** 2016-09-09

**Authors:** Min-Kyung Jang, Sunwoo Lee, Myeong Ho Jung

**Affiliations:** 1 Division of Longevity and Biofunctional Medicine, School of Korean Medicine, Pusan National University, Yangsan, Gyeongnam, South Korea; 2 GenomicWorks, Daejeon, South Korea; B.C. Cancer Agency, CANADA

## Abstract

In this study, we performed high throughput RNA sequencing at the preadipocyte (D0) and differentiated adipocyte (D7) stages of primary brown preadipocyte differentiation in order to characterize the transcriptional events regulating differentiation and function. Compared to the preadipocyte stage (D0), 6,668 genes were identified as differentially expressed genes (DEGs) with a fold change of ≥ 2.0 at the differentiated adipocyte stage (D7). Several adipogenic genes including peroxisome proliferator-activated receptor-γ (PPARγ) and CCAAT/enhancer-binding protein-α (C/EBPα), and Krüppel-like factor (KLF) family genes were differentially expressed at D0 and D7. Since KLF16 gene expression was downregulated at day 7 and its adipogenic function has not been characterized, we investigated its role in adipogenesis. Knockdown of KLF16 stimulated the differentiation of both brown and 3T3-L1 preadipocytes, and led to increased PPARγ expression. However, overexpression of KLF16 had opposite effects. Furthermore, KLF16 downregulated PPARγ expression in brown adipocytes and inhibited its promoter activity. These results indicate that KLF16 inhibits adipogenesis through downregulation of PPARγ expression.

## Introduction

There are two distinct types of adipose tissues, white adipose tissue (WAT) and brown adipose tissue (BAT). WAT plays a role in maintenance of energy homeostasis by storing lipids and releasing free fatty acids, as well as regulating whole-body insulin sensitivity by secretion of several adipocytokines [1]. BAT is a major site of nonshivering thermogenesis mediated by uncoupling of protein-1 (UCP1) in the mitochondria [2]. BAT can reduce diet-induced weight gain by increasing energy expenditure and improving insulin sensitivity [2]. Therefore, enhancing the differentiation and function of brown adipocytes may be therapeutically useful for treating obesity and its associated metabolic disorders. Consequently, understanding the molecular mechanisms regulating brown adipocyte formation should provide necessary insight for the development of novel treatment strategies against obesity.

The differentiation of brown and white preadipocytes into adipocytes (adipogenesis) occurs in several stages and is regulated by a cascade of key transcription factors [3, 4], including peroxisome proliferator-activated receptor-γ (PPARγ) and CCAAT/enhancer-binding protein-α (C/EBPα). PPARγ is a master regulator of adipogenesis of both types of preadipocytes, and induces expression of mature adipocyte phenotype-genes including the fatty acid binding protein (aP2), CD36. C/EBPα maintains PPARγ expression, and is required for differentiation of white preadipocytes but not brown preadipocytes. The master regulator of brown adipocyte differentiation is PRDM16 (PR-domain containing protein-16), which regulates PGC-1α (PPARγ-coactivator-1α), C/EBPβ, PPARγ, and PPARα, as well as induces expression of brown adipose-specific genes. Other transcription factors such as C/EBPδ, glucocorticoid (GR), cAMP response element binding protein (CREB), and sterol regulatory element-binding protein-1 (SREBP-1c) positively regulate adipogenesis. In addition, the Wnt/β-catenin pathway negatively regulates adipocyte differentiation. However, the transcription factors involved in differentiation of brown preadipocytes have not been defined yet.

In order to determine the factors and mechanisms regulating brown preadipocyte differentiation, we performed RNA-Seq to investigate the global gene expression patterns in primary (D0) and differentiated (D7) brown adipocytes. We determined that KLF16 is downregulated at D7, and is therefore a novel negative regulator of adipogenesis.

## Materials and Methods

### Antibody and reagent

The antibodies for KLF16 and PPARγ was purchased from Abcam (Cambridge, MA). Insulin, T3, indomethacin, isobutylmethylxanthine (IBMX) and dexamethasone were purchased from Sigma Aldrich (St. Louis, MO). Dulbecco’ Modified Eagles’ Medium (DMEM), fetal bovine serum (FBS), and penicillin/streptomycin were purchased from Life Technologies (Grand Island, NY)

### Isolation of brown preadipocytes and differentiation analysis

Primary brown preadipocytes were isolated from interscapular BAT of newborn mice, and immortalized with the retroviral plasmid, pBabepuro-large T, as previously described [5]. A pregnant C57BL/6J mouse which was purchased from Central Lab. Animal Inc. (Seoul, South Korea) was housed in polycarbonate cages under a12-h light-dark cycle at 21~23°C and 40~60% humidity until birth. Four mouse (1 day old) of either sex were used in this study. The mice was euthanized by CO_2_ exposure. The animal protocol used in this study was reviewed and approved by Pusan National University’s Institutional Animal Care and Use Committee (PNU–IACUC) in accordance with established ethical procedures and scientific care (approval number: PNU-2015-0940). Immortalized brown preadipocytes were cultured in DMEM containing 10% FBS and 1% penicillin/streptomycin. To differentiate brown preadipocytes, 90% confluent preadipocytes at day 0 were incubated in differentiation medium (20 nM insulin, 1 nM T3, 125 μM indomethacin, 500 μM IBMX and 0.5 μM dexamethasone), for 2 days, and then treated with differentiation medium supplemented with 20 nM insulin and 1 nM T3. 3T3-L1 preadipocytes obtained from the American Type Culture Collection (Manassas, VA, USA) were differentiated as described in brown adipocyte differentiation. At the indicated days, differentiated cells were stained by Oil Red O staining or subjected to qPCR.

### Total RNA preparation and Quantitative real time PCR (qPCR)

Total RNA was extracted using TRIZOL^®^ (Invitrogen, Paisley, Scotland) according to the manufacturer's instructions. DNA was digested using DNase I (Sigma). Total RNA was quantified by absorption of light at A260 using a spectrophotometer and the quality was checked by analyzing the A230/260 and A260/280 ratios (QIAxpert^®^, Qiagen, Hilden, Germany). cDNA was generated from 1 μg of total RNA using the GoScript^™^ Reverse Transcription System (Promega, Madison, WI) according to the manufacturer's protocol. PCR amplification was performed using gene specific primers. The primers used in this study are listed in S1 Table.

### Construction of transcriptome libraries and sequencing

Libraries were prepared according to Illumina’s protocol. Briefly, fragmentation buffer was added to samples in order to segment the mRNA into short fragments. Taking these short fragments as templates, random hexamer-primer was used to synthesize the first-strand cDNA. The second-strand cDNA was synthesized using buffer, dNTPs, RNaseH, and DNA polymerase I. Double-stranded cDNA was purified using the QiaQuick PCR extraction kit and resolved with EB buffer. Following synthesis of the 2^nd^ strand, end repair, addition of single A base, and adaptor ligation, cDNAs were connected to sequencing adaptors. The UNG enzyme was used to degrade the second-strand cDNA, and the product was purified using a MiniElute PCR Purification kit, following PCR amplification. The concentration of each library was measured by real-time PCR. Agilent 2100 Bioanalyzer was used for profiling the distribution of insert size. Constructed libraries were sequenced on an Illumina HiSeq^™^ 2000 according to the manufacturer’s instructions (Illumina Inc., USA); they were sequenced for 90 cycles. The HiSeq Control Software (HCS v1.1.37) with RTA (v1.7.45) was used to determine the management and execution of the experimental runs.

### Sequencing data analysis

Images generated by HiSeq^™^2000 analysis were converted to nucleotide sequences by base calling and stored in a FASTQ format. Clean reads were generated after filtering the dirty reads, which contained adaptors and unknown or low Phred quality scored-bases from raw reads. Several steps were performed for filtering the raw data as follows: 1) Reads containing > 9 N bases were removed. 2) Reads with > 36 low quality bases (<Q_20_) were removed. 3) Adaptor contamination was removed. 4) Duplication reads were removed. In addition, clean reads were mapped to reference mouse genome (mm10) and gene sequences using SOAP2aligner/SOAP2. Mismatches ≤ 5 bases were included in the alignment. Reads matched with reference rRNA sequences were also mapped and removed. To annotate gene expression, FPKM (fragments per kb per million reads) values of each gene were calculated and DEGs (differentially expressed genes) were extracted using the Agilent GeneSpring 7.3 program (Agilent Technologies, Palo Alto, CA, USA). A gene was considered differentially expressed if the difference in FPKM values between the two samples (D0 and D7) was ≥ 2.0-fold (i.e., log2 ratio > 1.0). For expression pattern analysis, hierarchical clustering analysis was performed using the Agilent GeneSpring 7.3 program. Furthermore, functional enrichment analysis was done using the Gene Ontology (GO) functional classification system (http://www.geneontology.org) and DAVID website (http://david.abcc.ncifcrf.gov/). All RNA-Seq data were deposited to the NCBI sequence read archive (SRA) database under accession number GSE73279.

### siRNA knockdown and plasmid DNA transfection

siRNAs for Fox and KLF16 genes used in this experiment were purchased from Sigma. The KLF 16 expression vector (pCMV-KLF16) was kindly provided by Dr. Urrutia. Brown preadipocytes and 3T3-L1 preadipocytes were transfected with siRNA or KLF 16 expression vector using the lipofectamine RNA iMAX reagent kit or lipofectamine LTX with PLUS Reagent kit (Invitrogen). The transfected cells were differentiated in differentiation medium 2 days after transfection.

### Reporter assay

The construction of mouse PPARγ promoter reporters was described previously [6]. For luciferase assays of PPARγ promoters, HepG2 cells or 3T3-L1 preadipocytes (2 × 10^4^ cells/well in a 96-well plate) were cotransfected with pCMV-KLF16 and PPARγ-Luc reporters and the luciferase activities were determined with a Dual-Glo Luciferase assay system kit (Promega).

### Statistical analysis

Data are expressed as the mean ± SEM. Statistically significant differences were determined by the two-tailed Student's t-test. For all statistical analyses, *p* values below 0.05 were considered significant.

## Results

### Adipogenesis of primary brown preadipocytes

**Fig 1:**
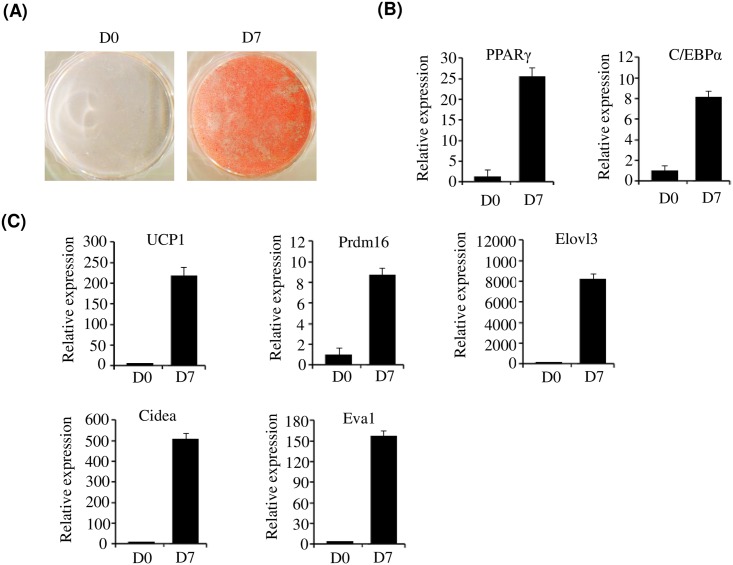
In vitro adipogenesis of primary brown preadipocytes. (A) Oil red O staining of primary brown preadipocytes. After inducing differentiation of primary brown preadipocytes, cells were stained with Oil Red O at D0 and D7 (B) Expression of PPARγ and C/EBPα was measured at D0 and D7 by qPCR. (C) Expression of brown adipocyte-specific genes, including UCP-1 and PRDM16, was measured at D0 and D7 by qPCR.

In order to get a global view of gene expression during adipogenesis of brown preadipocytes, RNA-Seq was performed to identify the genes involved and their function. RNA was isolated from primary cultured brown preadipocytes (D0) and differentiated adipocytes (D7). Differentiation of brown preadipocyte at D7 was confirmed by oil red O staining (Fig 1A) and measurement of PPARγ and C/EBPα levels by qPCR (Fig 1B). As shown in Fig 1, complete lipid accumulation and elevated expression of PPARγ and C/EBPα was observed. Furthermore, qPCR analysis demonstrated that brown specific genes including UCP1, PRDM16, elongation of very long-chain fatty acids 3 (Elovl3), cell death-inducing DFFA-like effector a (Cidea) and epithelial V-like antigen 1 (Eva1) were upregulated at D7 (Fig 1C). These results indicate that primary brown preadipocytes were fully differentiated at D7.

### RNA sequencing and identification of differentially expressed genes (DEG)

**Fig 2:**
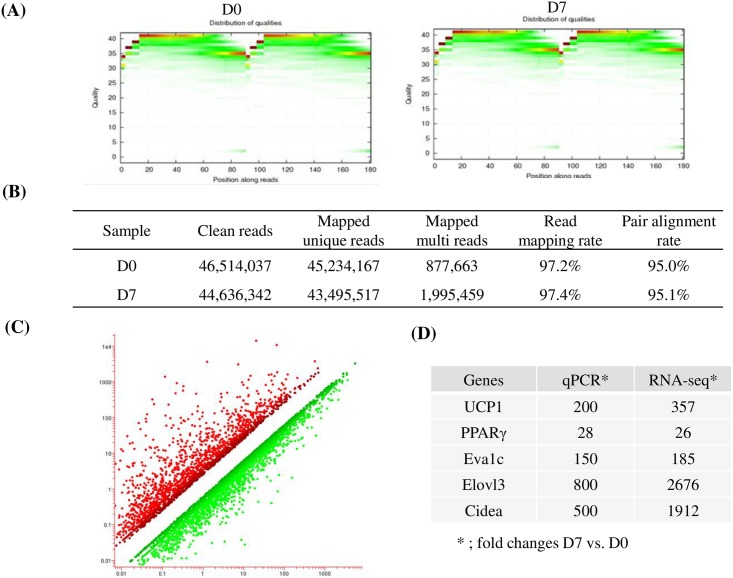
RNA-Seq analysis. (A) Quality distribution of bases along reads at D0 and D7 (B) Mapping result statistics at D0 and D7. (C) Clustering differentially expressed genes at D0 and D7. (D) Comparison of fold changes between RNA-Seq and qPCR.

mRNA fractions were isolated from total RNA obtained from brown preadipocytes at D0 and brown adipocytes at D7. An RNA-Seq library was generated using these mRNA fractions by RNA fragmentation, random hexamer-primed cDNA synthesis, linker ligation and PCR amplification. Each library was sequenced using an Illumina HiSeq 2000. To evaluate whether the RNA-Seq data was of sufficient quality for further analysis, we first assessed the global quality of the obtained reads (Fig 2A). After trimming, 46,514,037 (D0) and 44,636,342 (D7) clean reads were generated. Among these reads, 45,234,167 (D0) and 43,495,517 (D7) were mapped to the reference genome with mapping ratios of 97.2% and 97.4%, respectively (Fig 2B). This demonstrates the high quality of our sequencing datasets. Differentially expressed genes (DEG) were defined as those with a fold change of ≥ 2.0 on D7 compared to D0 (Fig 2C). Of these, 2,836 genes were upregulated and 3,832 genes were downregulated. To validate these DEGs, we compared the fold change of brown specific genes in RNA-Seq with those in the qPCR data. The results demonstrated similar expression patterns between RNA-Seq and qPCR, thus validating the RNA-Seq data (Fig 2D).

### The role of KLF16 in the regulation of adipogenesis

The regulation of preadipocyte differentiation has been extensively studied, and an elaborate network of transcription factors culminating in the expression of PPARγ and C/EBPα has been partially defined [3, 4]. We identified several known adipogenic genes that were differentially expressed at D0 and D7 (data not shown). The upregulated genes included positive adipogenic regulators such as PPARγ and C/EBPα, whereas the downregulated genes included negative adipogenic regulators such as GATA2 and Wnt5a. The master adipogenic transcription factors, PPARγ and C/EBPα, are regulated by many other transcriptional regulators, including members of the Fox and KLF family. Interestingly, we observed that Fox (Table 1) and KLF (Table 2) genes were differentially expressed.

**Table 1:** Fox family that differentially expressed at D7.

Gene symbol	Gene Name	Fold
Foxl2	forkhead box l2	2.02
Foxe1	forkhead box e1	4.61
Foxd3	forkhead box d3	4.68
Foxo1	forkhead box o1	4.05
Foxo6	forkhead box o6	27.4
Foxa3	forkhead box a3	2.32
Foxa2	forkhead box a2	2.26
Foxf1	forkhead box f1	0.12
Foxm1	forkhead box m1	0.21
Foxc2	forkhead box c2	0.28
Foxs1	forkhead box s1	0.32
Foxp1	forkhead box p1	0.34
Foxp4	forkhead box p4	0.41
Foxf2	forkhead box f2	0.46
Foxk2	forkhead box k2	0.50

**Table 2:** KLF family that differentially expressed at D7.

Gene symbol	Gene Name	Fold
Klf13	Kruppel-like factor 13	2.49
Klf5	Kruppel-like factor 5	2.29
Klf15	Kruppel-like factor 15	10.2
Klf9	Kruppel-like factor 9	5.05
Klf16	Kruppel-like factor 16	0.28
Klf2	Kruppel-like factor 2	0.40
Klf12	Kruppel-like factor 12	0.42
Klf8	Kruppel-like factor 8	0.43
Klf14	Kruppel-like factor 14	0.47

We verified the expression of KLF and Fox genes expressed differentially in RNA-seq data. qPCR showed that similar expression patterns between RNA-Seq and qPCR was observed. (S1 Fig).

**Fig 3:**
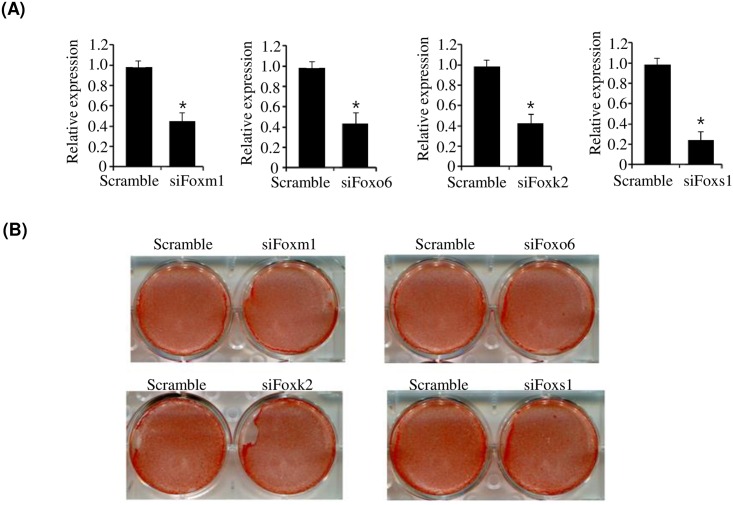
Effect of Foxs (Foxm1, Foxo6, Foxk2, and Foxs1) on the adipogenesis of brown preadipocytes. (A) Expression of Foxm1, Foxo6, Foxk2, and Foxs1 in siRNA-transfected primary brown preadipocytes. Primary brown preadipocytes were transfected with siRNA for Foxm1, Foxo6, Foxk2, and Foxs1, and their expression was measured by qPCR (B) Oil red O staining of brown preadipocytes transfected with siRNAs for Foxm1, Foxo6, Foxk2, and Foxs1. Primary brown preadipocytes were transfected with siRNAs for Foxm1, Foxo6, Foxk2, and Foxs1, and were then differentiated in differentiation medium for 7 days. Lipid accumulation was assessed by Oil Red O staining. qPCR data are presented as means±SEM from three independent experiments. *P< 0.05 vs. scramble.

In the Fox family, FoxC2, which was downregulated at D7, has been reported to inhibit adipocyte differentiation [7], whereas FoxO1, which was upregulated at D7, has been reported to activate adipogenesis [8]. However, the role of FoxO6, FoxM1, FoxK2, and FoxS1, which were also differentially expressed at D7, remains unknown in adipogenesis. Therefore, we investigated whether these Fox family members regulate adipogenesis. Using the siRNA knockdown technique, we examined adipogenesis in Fox-deficient 3T3-L1 cells. As shown in Fig 3A, siRNA knockdown successfully reduced the expression of each Fox gene. However, knockdown of each Fox gene evaluated did not affect adipocyte differentiation (Fig 3B).

**Fig 4:**
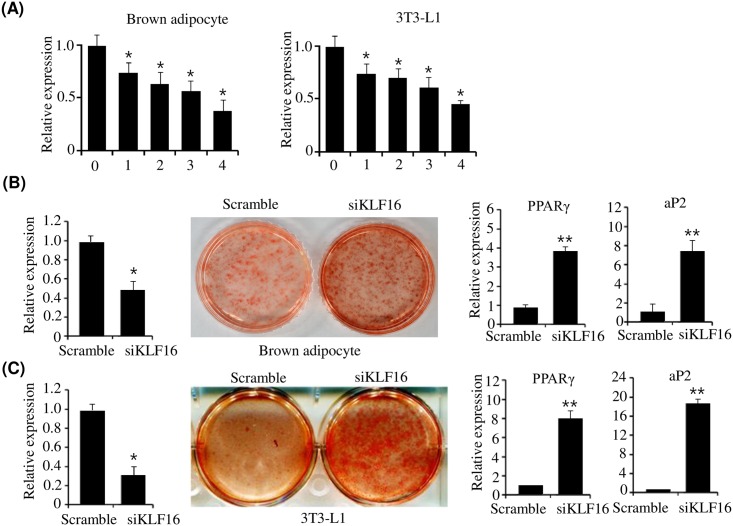
Effect of KLF16 on adipogenesis. (A) Expression of KLF16 during differentiation of primary brown preadipocytes or 3T3-L1 preadipocytes. Primary brown preadipocytes or 3T3-L1 preadipocytes were differentiated in differentiation medium and the expression of KLF16 was determined at the indicated times by qPCR. qPCR data are presented as means±SEM from three independent experiments. *P< 0.05 vs. D0. (B) Knockdown of KLF16 stimulates adipogenesis of brown preadipocytes. Primary brown preadipocytes were transfected with KLF16 siRNA and were differentiated in differentiation medium for 7 days. KLF16 knockdown was confirmed in KLF16 siRNA-transfected brown preadipocytes by qPCR. Adipocyte differentiation was assessed by oil red O staining and expression of PPARγ and aP2 on D0 and D7. (C) Knockdown of KLF16 stimulates adipogenesis of 3T3-L1 preadipocytes. 3T3-L1 preadipocytes were transfected with KLF16 siRNA and were differentiated in differentiation medium for 7 days. KLF16 knockdown was confirmed in KLF16 siRNA-transfected 3T3-L1 preadipocytes by qPCR. Adipocyte differentiation was assessed by oil red O staining and expression of PPARγ and aP2 on D0 and D7. qPCR data are presented as means±SEM from three independent experiments. *P< 0.05, **P<0.01 vs. scramble.

Next, we investigated the role of KLF family genes, which were also differentially expressed. The upregulated genes, KLF 5, 9, and 15, have been reported to activate adipogenesis [9–11], whereas downregulated KLF2 inhibits adipogenesis [12]. The expression pattern of these KLF proteins in our analysis was consistent with negative or positive adipogenesis regulation. However, the function of KLF16, a recently discovered KLF family member that was downregulated at D7, remains largely uncharacterized. Therefore, we investigated the role of KLF16 in adipogenesis. To this end, we first confirmed the expression of KLF16 during adipogenesis of brown preadipocyte and white adipocyte 3T3-L1 cells. KLF16 was highly expressed in preadipocyte cells, but gradually decreased after adipogenic induction, whose expression at D0 and D7 was consistent with the RNA-Seq data (Fig 4A). These results suggest that KLF16 may negatively regulate adipogenesis. To address whether KLF16 plays a role in the adipogenesis of brown preadipocytes, we investigated adipogenesis in KLF16-deficient brown preadipocytes. Knockdown of KLF16 using siRNA was confirmed by qPCR (Fig 4B). Knockdown of KLF16 greatly increased the adipogenesis of brown preadipocytes compared with control siRNA (Fig 4B). In addition to lipid accumulation, the expression of adipogenic marker genes such as PPARγ and aP2 also increased in KLF16-knockdown cells (Fig 4B). The stimulation of adipogenesis through knockdown of KLF16 was also observed in KLF16-knockdown 3T3-L1 cells (Fig 4C).

**Fig 5:**
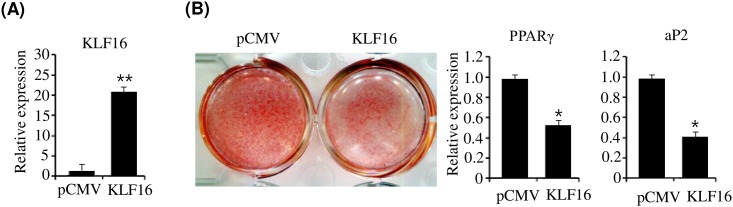
Overexpression of KLF16 inhibits adipogenesis of primary brown preadipocytes. Primary brown preadipocytes were transfected with pCMV-KLF16 and were differentiated in differentiation medium for 7 days. (A) KLF16 expression was determined in KLF16-transfected brown preadipocytes by qPCR. qPCR data are presented as means±SEM from three independent experiments. **P< 0.05 vs. control vector (pCMV). (B) Adipocyte differentiation was assessed by Oil Red O staining and expression of PPARγ and aP2 on D0 and D7. qPCR data are presented as means±SEM from three independent experiments. *P< 0.05 vs. control vector (pCMV).

Furthermore, to confirm the effect of KLF16 on adipogenesis, we evaluated adipogenesis in KLF16-overexpressed brown adipocytes. qPCR showed that KLF16 was overexpressed in brown preadipocytes (Fig 5A). However, overexpression of KLF16 inhibited adipogenesis of brown preadipocytes, which was determined by Oil Red O staining and expression of adipogenic genes (Fig 5B). Taken together, these results indicate that KLF16 inhibits adipogenesis of both brown and white preadipocytes.

### KLF16 represses PPARγ expression in adipocytes

**Fig 6:**
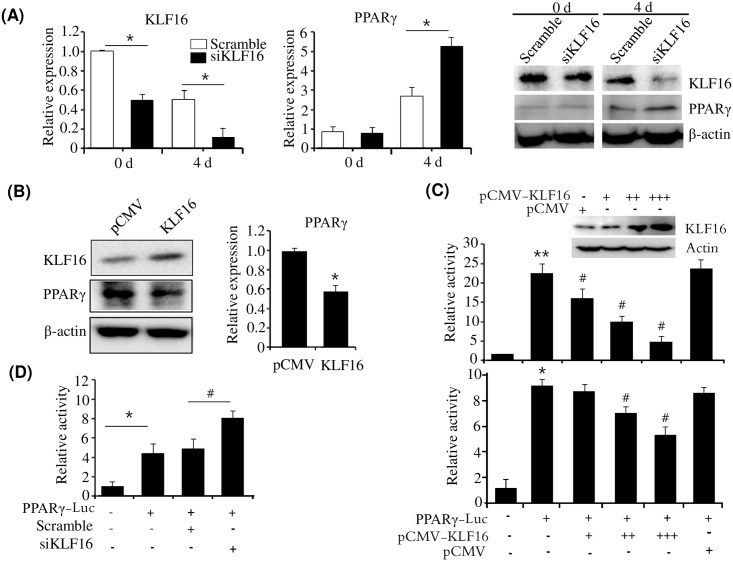
KLF16 inhibits PPARγ expression. (A) Knockdown of KLF16 led to increased PPARγ expression. Primary brown preadipocytes were transfected with KLF16 siRNA and were differentiated in differentiation medium. Expression of KLF16 and PPARγ was determined at D0 and D4 by western blot and qPCR. qPCR data are presented as means±SEM from three independent experiments. *P< 0.05 vs. scramble at D0 and D4, respectively. (B) Overexpression of KLF16 repressed PPARγ expression. Brown adipocytes were transfected with pCMV-KLF16, and the expression of KLF16 and PPARγ was determined by western blot and qPCR, respectively. qPCR data are presented as means±SEM from three independent experiments. *P< 0.05 vs. control vector (pCMV). (C) Overexpression of KLF16 inhibited the promoter activity of PPARγ. HepG2 cells (upper) or 3T3-L1 preadipocytes (bottom) were cotransfected with a -2.6 Kb PPARγ-Luc reporter and pCMV-KLF16, and luciferase activities were determined. Data are presented as means±SEM from three independent experiments. *P< 0.05 **P< 0.01 vs. control vector (pGL3). ^#^*P*< 0.05 vs. PPARγ-Luc reporter. (D) Knockdown of KLF16 increased the promoter activity of PPARγ. 3T3-L1 preadipocytes were cotransfected with a -2.6 Kb PPARγ-Luc reporter and KLF16 siRNA, and luciferase activities were determined. Data are presented as means±SEM from three independent experiments. *P< 0.05 vs. control vector (pGL3). ^#^*P*< 0.05 vs. PPARγ-Luc reporter & scramble.

PPARγ is a master regulator of adipogenesis. To determine whether PPARγ is implicated in KLF16-mediated inhibition of adipogenesis, we determined the expression of PPARγ in KLF16-knockdown brown adipocytes. Inhibition of KLF16 expression via siRNA led to increased expression of PPARγ in KLF16-deficient brown adipocytes at D4 (Fig 6A), suggesting that KLF16 downregulates PPARγ expression. To further confirm the downregulation of PPARγ by KLF16, we investigated PPARγ expression in KLF16 overexpressed brown adipocytes. Western blot analysis showed that KLF16 was overexpressed in brown adipocytes (Fig 6B). However, overexpression of KLF16 efficiently repressed PPARγ expression in brown adipocytes, which was revealed by western blot and qPCR analysis (Fig 6B). To determine whether the repression of PPARγ by KLF16 is regulated at the transcriptional level, we examined the effect of KLF16 on the promoter activity of PPARγ. Overexpression of KLF16 inhibited the promoter activity of PPARγ in a dose dependent manner in HepG2 cells or 3T3-L1 preadipocytes (Fig 6C). Furthermore, to confirm the repressive effect of KLF16, we measured the promoter activity of PPARγ in KLF16-knockdown 3T3-L1 preadipocyte. As shown in Fig 6D, KLF16 knockdown using siRNA increased promoter activity of PPARγ-Luc in 3T3-L1 preadipocytes. These results suggest that KLF16 inhibits adipogenesis through downregulation of PPARγ by repressing its promoter activity.

**Fig 7:**
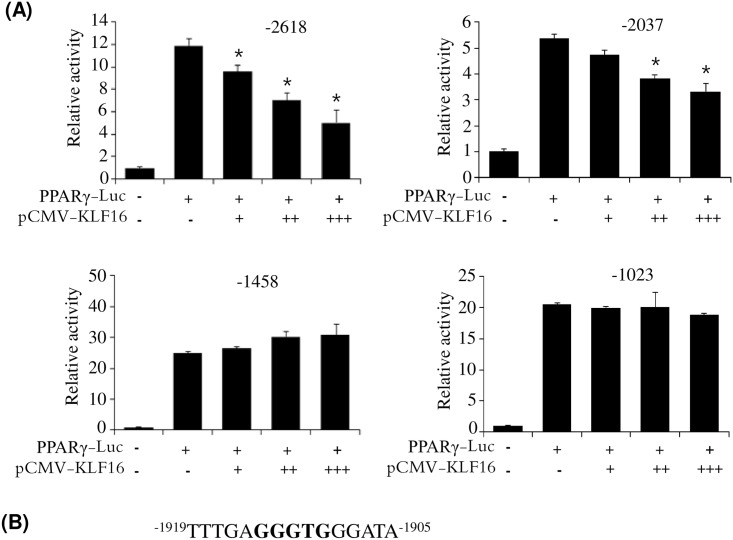
KLF16 binding site is between −1914 and −1910 in the PPARγ promoter. (A) 5′ serial deletion reporters of PPARγ promoter were transfected into HepG2 cells with pCMV-KLF16, and then luciferase activities were measured. Data are presented as means±SEM from three independent experiments. *P< 0.05 vs. PPARγ-Luc reporter. (B) Putative KLF16 binding site between −1914 and −1910.

To assess which KLF binding element is critical for repression of PPARγ expression by KLF16, we measured the effects of ATF3 on promoter activities of several 5′-deleted promoters. As shown in Fig 7, deletions between −2618 and −2037 in the promoter retained the KLF16-mediated repressive effect on promoter activity. However, deletions between −2037 bp and −1458 bp in the promoter significantly abolished the repressive effect by KLF16, suggesting that the KLF16-responsive motif is located in the region between −2037 and −1458. Within this region, a candidate KLF binding motif (5′-CACCC-3′) is present between −1914 and −1910, and could be involved in KLF16-mediated repression of PPARγ expression.

## Discussion

Recently, BAT has received significant attention as a therapeutic target for treating obesity and obesity-associated metabolic diseases and complications such as type 2 diabetes [2, 13]. This is because BAT dissipates excess lipids as heat through adaptive thermogenesis. Augmenting BAT function and mass is an attractive therapeutic approach to treating obesity and its associated metabolic disorders. Therefore, understanding the molecular events regulating brown adipocyte formation and function can lead to the successful treatment of obesity.

To investigate the transcriptional changes during the adipogenesis of brown preadipocytes, we performed RNA-Seq at different stages of primary brown preadipocyte differentiation, specifically pre-adipocytes (D0) and mature adipocytes (D7). RNA-Seq analysis identified 6,668 genes, which were differentially expressed. Among them, 2,836 were upregulated at D7, whereas 3,832 were downregulated. BAT is a thermogenic organ that metabolizes both fatty acids and glucose to produce heat through uncoupling protein 1 (UCP1), which uncouples the mitochondrial proton gradient from ATP synthesis to generate heat. The GO of the differentially expressed genes demonstrated that genes associated with thermogenesis, energy generation, oxidative phosphorylation, and fatty acid oxidation were upregulated, suggesting that differentiated BAT at D7 was functional. We also observed a number of adipogenesis-associated genes in the DEGs. While the master regulators of adipogenesis, PPARγ and C/EBPα, were upregulated, GATA2 and Wnt5a were downregulated at D7. These results were consistent with previous studies. In order to identify a noble regulator of adipogenesis among the DEGs, we first selected the Fox family of proteins (Foxm1, Foxk2, Foxo6, and FoxS1), which were differentially expressed during adipogenesis, because some Fox genes have been reported to regulate adipogenesis [10, 11]. Here, we analyzed the adipogenesis of Fox-knockdown brown preadipocytes. However, knockdown of the different Fox proteins by siRNA did not influence the adipogenesis of brown preadipocytes. Additionally, we examined whether the differentially expressed KLF family of proteins regulate adipogenesis. The family of Kruppel-like factor (KLF) proteins derived from *Drosophila* embryonic pattern regulator protein Kruppel, consists of 17 members containing a C2H2 zinc finger at the C-terminal that regulates cell proliferation, differentiation, apoptosis, and development [14, 15]. These proteins regulate gene expression by binding to GC rich sequences of gene promoters, the GC/GT boxes. Recently, it has been demonstrated that several KLFs are involved in adipogenesis [16]. KLF 4, 5, 6, 9, and 15 promote adipogenesis, whereas KLF2, 3, and 7 inhibit adipogenesis. We observed that KLF4, KLF5, KLF6, and KLF15 were positive regulators of adipogenesis, whereas KLF3, KLF2, and KLF7 were negative regulators. Based on our RNA-Seq data, the positive regulators KLF5, 15, and 9 were upregulated at D7, whereas the negative regulator KLF2 was downregulated at D7. However, KLF16, the gene expression of which was downregulated at D7, has not been reported to play a role in adipogenesis until now. qPCR analysis demonstrated that the expression of KLF16 was decreased during adipocyte differentiation, consistent with the RNA-Seq data. This suggests that KLF16 may be an inhibitor of adipogenesis. siRNA-mediated knockdown of KLF16 stimulated the adipogenesis of both brown preadipocytes and 3T3-L1 preadipocytes. In contrast, overexpression of KLF16 inhibited adipogenesis, which demonstrated that KLF16 is a negative regulator of adipogenesis.

It has been reported that KLFs influence adipogenesis by regulating the expression of PPARγ, C/EBPα, and C/EBPβ [9–12, 17, 18]. In this study, we investigated the mechanism by which KLF16 inhibits adipocyte differentiation. Inhibition of KLF16 during adipocyte differentiation resulted in stimulation of PPARγ expression. Conversely, overexpression of KLF16 inhibited PPARγ expression, suggesting that KLF16 may downregulate PPARγ expression, which may contribute to inhibition of adipocyte differentiation. To validate this result, we investigated the effect of KLF16 on the promoter activity of PPARγ. Overexpression of KLF16 resulted in suppression of PPARγ promoter activity in a concentration-dependent manner. KLFs bind to the GC- and CNCCCC-boxes of the promoter region of their target genes [5]. Several KLF-binding elements are present in the PPARγ promoter. The promoter assays of 5′-deleted PPARγ promoter reporters indicated that KLF binding motif (5′-GGGTG-3′) between −1914 and −1910 is a potential KLF16 binding site on the PPARγ promoter.

In conclusion, RNA-Seq analysis identified KLF16 as a novel PPARγ target gene. We show that it represses PPARγ expression, resulting in inhibition of adipogenesis.

## Supporting Information

S1 Fig Validation of the expression of genes of Fox and KLF family. Expression of genes of Fox and KLF family was measured at D0 and D7 by qPCR.
(TIF)

S1 Table List of primers for qPCR. (DOCX)
