# Supplementary figures and images for: RNA-Seq Analysis Reveals a Negative Role of KLF16 in Adipogenesis
Source: PLoS One. 2016 Sep 9;11(9):e0162238. doi: 10.1371/journal.pone.0162238 (PMC5017575; doi:10.1371/journal.pone.0162238)

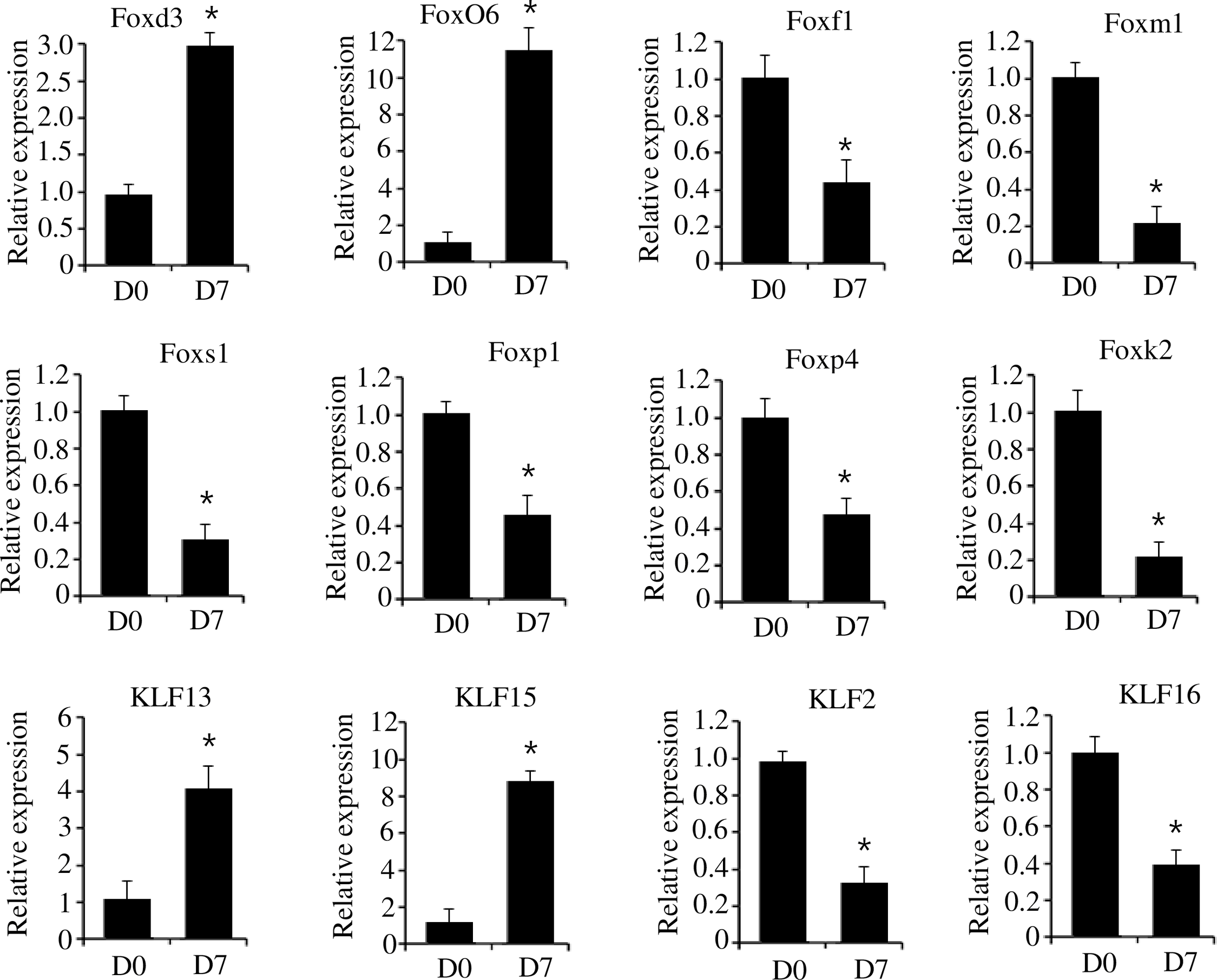

Supplement: S1 Fig — Expression of genes of Fox and KLF family was measured at D0 and D7 by qPCR. (TIF) [file pone.0162238.s001.tif]
