# Supplementary material for: RNA-Seq Analysis Reveals a Negative Role of KLF16 in Adipogenesis
Source: PLoS One. 2016 Sep 9;11(9):e0162238. doi: 10.1371/journal.pone.0162238 (PMC5017575; doi:10.1371/journal.pone.0162238)
Supplement: S1 Table — (DOCX) [file pone.0162238.s002.docx]

S1 Table. List of primers for q- PCR

| Gene | Forward primer | Reverse primer |
| --- | --- | --- |
| PPARγ | GTGCCAGTTTCGATCCGTAGA | GGCCAGCATCGTGTAGATGA |
| C/EBPα | CAAGAACAGCAACGAGTACCG | GTCACTGGTCAACTCCAGCAC |
| aP2 | ACACCGAGATTTCCTTCAAACTG | CCATCTAGGGTTATGATGCTCTTCA |
| UCP-1 | ACTGCCACACCTCCAGTCATT | CTTTGCCTCACTCAGGATTGG |
| PRDM16 | CAGCACGGTGAAGCCATTC | GCGTGCATCCGCTTGTG |
| KLF16 | GTGTACCAAGCGGTTCACC | CAGGTCGTCGCAGGAGTTC |
| Cidea | TGCTCTTCTGTATCGCCCAGT | GCCGTGTTAAGGAATCTGCTG |
| Elovl3 | TCCGCGTTCTCATGTAGGTCT | GGACCTGATGCAACCCTATGA |
| Eva1 | GTCCCAACCAGACCATCAAC | CTCCATCTTGCTCTGGAAGC |
| Foxm1 | CTGATTCTCAAAAGACGGAGGC | TTGATAATCTTGATTCCGGCTGG |
| Foxo6 | TCATGGACAGTGACGAAATGG | ACCCAGCTCTGGTTAGGGG |
| Foxk2 | TGCTGTCTGCTCACTCCAGT | GACGGGCTGACTAGAGAGAGG |
| Foxs1 | CTATCCAGAGTTCACCGGGTC | GTTATGGCGGATGCTGTTTTG |
